# Setting the Stage: Measure Selection, Coordination, and Data Collection for a National Self-Management Initiative

**DOI:** 10.3389/fpubh.2014.00206

**Published:** 2015-04-27

**Authors:** Kristie P. Kulinski, Michele Boutaugh, Matthew Lee Smith, Marcia G. Ory, Kate Lorig

**Affiliations:** ^1^National Council on Aging, Washington, DC, USA; ^2^Administration on Aging, Administration for Community Living, Atlanta, GA, USA; ^3^Department of Health Promotion and Behavior, College of Public Health, The University of Georgia, Athens, GA, USA; ^4^Department of Health Promotion and Community Health Sciences, School of Public Health, Texas A&M Health Science Center, College Station, TX, USA; ^5^Stanford Patient Education Research Center, Department of Medicine, Stanford School of Medicine, Palo Alto, CA, USA

**Keywords:** chronic disease self-management, evidence-based program, data collection, intervention planning

## Abstract

This paper describes the history and rationale behind the development of a centralized data collection system for the national rollout of the Chronic Disease Self-Management Program (CDSMP) through the American Recovery and Reinvestment Act of 2009 Communities Putting Prevention to Work*:* CDSMP initiative. In addition to justifying the need for solutions to the burgeoning burden of chronic disease in the United States, this paper provides details about CDSMP and related self-management education programs, including their structure, facilitator training, and effectiveness. These topics set the stage for the processes and procedures to create and manage the database for use at the national, state, and local levels. Furthermore, this paper describes the processes related to selecting variables, coordinating data collection, and utilizing data to inform research and policy.

## Rationale

As more and more evidence-based interventions are being funded as multi-site programs by both federal agencies and major foundations, there is a growing need for uniform measures and protocols as well as centralized data collection systems. This paper describes one such data system developed in response to the national rollout of the Chronic Disease Self-Management Program (CDSMP) through the American Recovery and Reinvestment Act of 2009 Communities Putting Prevention to Work*:* CDSMP initiative (ARRA CDSMP) (1). In addition to describing the creation and management of the database used in this effort, we will discuss its uses at the national, state, and local levels as well as its utility for informing policy and research. Additionally, this paper will provide the necessary background for those wanting to understand the rationale behind this national initiative in terms of the burden of chronic conditions among older Americans and self-management (SM) as a core requirement for dealing with such conditions. As a case example, the processes related to selecting variables, developing a centralized data collection system, training, and managing data will be described for this grand-scale translational rollout of an evidence-based program.

## Background

### Burden of chronic conditions

Chronic conditions have become endemic in the United States, with older adults bearing the greatest burden. Approximately 36% of adults age 18–34 have a chronic condition, compared to nearly 92% in the population aged 65 and over (2). This same trend is observed with regard to multiple chronic conditions, with a range of 14% among the population aged 18–34 to nearly 77% in the older adult population (2). Among Medicare beneficiaries, the most common chronic conditions include high blood pressure (58%), high cholesterol (45%), heart disease (31%), arthritis (29%), and diabetes (28%) (3).

Older adults with chronic conditions face a number of barriers in terms of coping with their illness and optimizing their health, which include lack of social support, low skill levels for symptom management, and low confidence in their abilities to manage their conditions (self-efficacy) (4). Increasingly, SM is being heralded as a key component in the improvement of health outcomes associated with chronic disease. According to the Institute of Medicine, SM is defined as “the tasks that individuals must undertake to live well with one or more chronic conditions.” (5) Research demonstrates the positive impact of SM programs on these tasks, which include having the confidence to deal with the medical, role, and emotional management of their conditions (5, 6).

### The chronic disease self-management program

The CDSMP is perhaps the best known SM intervention (7). It was developed at Stanford University and is a peer-led, community-based intervention that helps individuals with chronic conditions learn skills and gain the confidence to manage and improve their health (7). The program focuses on challenges that are common to individuals living with any chronic condition, such as problem solving, decision making, symptom management, nutrition, exercise, medication use, emotions, and communicating with health care professionals. In addition to the standard CDSMP, Stanford offers a comprehensive suite of chronic disease self-management education (CDSME) programs, with disease-specific variants for people living with diabetes, chronic pain, HIV/AIDS, cancer survivors, and arthritis. Most of these also have culturally appropriate Spanish versions. The programs are available in over 30 countries and 25 languages.

Led by a pair of trained facilitators, many of whom also have chronic health conditions, these small, highly interactive workshops meet once a week for six consecutive weeks. During each 2.5-hour session, 10–15 participants focus on building the skills they need to manage their conditions. Fostering participant self-efficacy is at the core of the intervention, achieved through techniques such as skills mastery, peer modeling, reinterpretation of physiological symptoms, and social persuasion. Workshops are highly participative, with mutual success and support building participants’ confidence in their ability to manage their health and maintain active, fulfilling lives. Participants create a weekly action plan and try new behaviors such as exercise monitoring. Each session includes an opportunity for feedback about progress and discussion of challenges. Table 1 provides an overview of the topics and activities covered during each workshop session.

**Table 1:** **CDSMP workshop overview by session**.

	Week 1	Week 2	Week 3	Week 4	Week 5	Week 6
Overview of self-management and chronic health conditions	✓					
Making an action plan	✓	✓	✓	✓	✓	✓
Relaxation/cognitive symptom management	✓		✓	✓	✓	✓
Feedback/problem solving		✓	✓	✓	✓	✓
Difficult emotions		✓	✓			
Fitness/exercise		✓	✓			
Better breathing			✓			
Fatigue			✓			
Eating well				✓		
Advance directives				✓		
Communication				✓		
Medications					✓	
Making treatment decisions					✓	
Depression					✓	
Informing the health care team						✓
Working with your health care professional						✓
Future plans						✓

#### Workshop facilitator training and infrastructure

The program uses a train-the-trainer model consisting of Lay Leaders, Master Trainers, and T-Trainers (8). Lay Leaders can facilitate CDSMP workshops but cannot train others. They complete a structured training and must facilitate at least one workshop in the following year. Master Trainers can facilitate CDSMP workshops as well as train new Lay Leaders. As with CDSMP Lay Leaders, Master Trainers participate in a systematized training. After training, they must facilitate at least two CDSMP workshops within one year and conduct a Lay Leader training within 18 months. Finally, T-Trainers are authorized to facilitate workshops, train new Lay Leaders, and train new Master Trainers. This role involves the completion of an apprenticeship with a Stanford staff T-Trainer. Additionally, they must have facilitated at least three Lay Leader trainings prior to their apprenticeship, co-lead a Master Trainer training within 12 months of completing the apprenticeship, and conduct a Master Trainer training every two years.

#### Intervention effectiveness

The Chronic Disease Self-Management Program has been extensively evaluated through randomized controlled trials (9, 10). Workshop participants experience significant improvements across several domains, including physical activity, symptom management, communication with physicians, and general health. Additionally, the original research demonstrated that CDSMP participants spend fewer days in the hospital, as well as a trend toward fewer outpatient visits and hospitalizations (10).

Further cementing the value of CDSMP, the program has been successfully translated for implementation in a variety of community settings worldwide, with participants reporting results similar to the original research. A recent United States-based *National Study of CDSMP* encompassed over 1000 participants drawn from 145 workshops in 17 states (11). Sociodemographic, health status, and behavioral data were collected at baseline, 6, and 12 months, yielding a number of positive, significant improvements (12, 13). When aligned with the Institute for Healthcare Improvement’s Triple Aim (13, 14), the following results are particularly noteworthy: better health – improvement in self-reported health, less depression, and better quality of life; *better care –* improved communication with physicians, medication compliance, and health literacy; and *lower health cost –* more than $360 per person net savings after factoring in program costs (15). In addition to improving participant health and decreasing health care costs, the outcomes of this national study reinforce that CDSMP has been effectively translated from research to practice throughout the country.

## National Initiatives Supporting CDSMP Implementation

Over the past decade, community-based implementation of CDSMP and its variants have received broad support through funding from federal agencies [e.g., the Administration on Aging (AoA), a program division within the Administration for Community Living, and the Centers for Disease Control and Prevention (CDC)], foundations (e.g., Atlantic Philanthropies, Archstone Foundation, Robert Wood Johnson Foundation, and Health Foundation of South Florida), and health care providers (e.g., Kaiser Permanente, Group Health Cooperative, and Dignity Health). Specific to the aging services network, AoA has supported states and community organizations in their efforts to develop infrastructure, workforce, and capacity to deliver CDSMP and other evidence-based programs. Since 2006, AoA has provided three major competitive grant programs to states to support dissemination of evidence-based programs. The 2006–2012 *Evidence-Based Disease and Disability Prevention Program* (EBDDP) grants were awarded to 24 states to support dissemination of CDSMP and evidence-based physical activity, fall prevention, nutrition, and behavioral health programs. The national program infrastructure was greatly expanded with the 2010–2013 *American Recovery and Reinvestment Act Communities Putting Prevention to Work: CDSMP* (ARRA CDSMP) grants awarded to 45 states, the District of Columbia, and Puerto Rico. The administration’s current 2012–2015 *Empowering Older Adults and Adults with Disabilities through Chronic Disease Self-Management Education Programs* grant program, financed by the Prevention and Public Health Fund (PPHF), provides support to 22 states. Both the ARRA CDSMP and the PPHF grant programs have focused on not only chronic disease SM programs, including the generic CDSMP, but also programs developed for specific chronic conditions (arthritis, diabetes, HIV/AIDs, and chronic pain), for Spanish-speaking cultures, and in an online format. Table 2A highlights AoA funding history, although as previously noted a number of federal and other sources of funding have also supported these programs.

**Table 2:** **Support, data requirements, and rationale for AoA-CDSMP initiatives**.

**A: EVOLUTION OF AoA-SUPPORTED CDSMP INITIATIVES**		
Year	Initiative	Reach
2003	Evidence-Based Prevention Program for the Elderly Model Communities Project	14 Communities
2006	Evidence-Based Disease Prevention and Disability Program	16 States
2006–2007	Evidence-Based Disease Prevention and Disability Program (funding made available to 24 states by AoA, plus three states funded by Atlantic Philanthropies)	27 States
2010	American Recovery and Reinvestment Act Communities Putting Prevention to Work: Chronic Disease Self-Management Program	47 States/Territories
2012	2012 Prevention and Public Health Funds: Empowering Older Adults and Adults with Disabilities through Chronic Disease Self-Management Education Programs	22 States

**B: AoA GRANTEE DATA COLLECTION REQUIREMENTS**		
**Data type**	**Elements collected**	

Workshop Information	Host organization and implementation site name/location, workshop leader names, workshop start/end dates, use of orientation session, workshop type, workshop language	
Participant Information	Date of birth, ZIP Code, sex, race, ethnicity, chronic conditions, caregiver status, disability status, number of people in household, education level	
Attendance	Sessions attended by participant	
Organization Data	Organization type with regard to host organization and implementation site (list includes area agency on aging, county health department, health care organization, faith-based organization, workplace, residential facility, and library)	

**C: RATIONALE FOR SELECTING DATA TYPES**		
**Data type**	**Rationale**	

Workshop Information	Map delivery infrastructure, identify type of workshop offered, identify diversity of languages, monitor start/end dates and number of workshop leaders as proxies for fidelity	
Participant Information	Accurately describe participant population, ensure adequate reach to target population, monitor demographic elements that serve as proxies for health status and vulnerability (race/ethnicity, chronic conditions, caregiver status, disability status, education level, etc.)	
Attendance	Track number of sessions attended by participant to determine completer status, identify organization and state successes/challenges with participant retention	
Organization Data	Identify types of organizations involved in program delivery, monitor increase in delivery capacity and geographic reach	

## Data Collection

### Selecting standardized measures

The collection of standardized performance monitoring data has been a critical component of each of the aforementioned AoA initiatives. While the specific measures collected have evolved over time, the data collected by AoA grantees and their partners can be grouped within four categories: (1) workshop information; (2) participant information; (3) attendance; and (4) organization data. The current standardized measures, which were approved through the Office of Management and Budget (OMB) Paperwork Reduction Act, are listed in Table 2B.

### Rationale for uniform data collection and monitoring

Data elements for the ARRA CDSMP initiative were carefully and purposively chosen with the intent of balancing the critical need to monitor program operations and participant accrual with the desire to minimize data collection and reporting burden on program deliverers and participants (see Table 2C). Considering the myriad studies reinforcing the effectiveness of the program in the community when delivered with fidelity to the original model (6, 12), emphasis was placed on collecting reach data versus additional outcome data. Moreover, grantees and their partners were encouraged to invest their limited resources in program delivery, infrastructure, and sustainability to ensure ongoing access to CDSMP as opposed to engaging in costly outcomes measurement.

At the federal level, there is a strong emphasis on accountability and transparency to ensure that funds are being spent properly and the desired reach and impact are being achieved. Therefore, the uniform collection of appropriate measures ensures that due diligence is performed in this regard. For example, an overarching goal of the ARRA CDSMP initiative was to reach 50,000 program completers (i.e., those participants who attend four or more of the six workshop sessions), with a particular emphasis on engaging vulnerable and disadvantaged older adults (16). Because detailed attendance information was collected on each participant, it was easy to determine how many completers were reached. This attendance information was especially important because outcome measures were not collected in this initiative, thus workshop attendance served as a proxy variable indicating that participants received an adequate intervention dose. Additionally, because outcomes were not directly measured, demographic variables such as date of birth, living alone status, racial and ethnic status, education level, and number of chronic conditions served as proxies for health status and vulnerability. Collecting this participant and attendance information was deemed important for informing national and state leadership as to whether or not the target population was being reached/served by intervention workshops.

### Data collection tools, coordination, and processes

Timely and efficient collection and reporting of programmatic data are critical to ensure the success and value of the national database. The following OMB-approved data collection forms accompany the national database: (1) Workshop Information Cover Sheet; (2) Attendance Log; (3) Participant Information Survey; and (4) Organization Data Form.

At a local level, workshop leaders complete the Workshop Information Cover Sheet. They are also responsible for using the Attendance Log to track participation at each session. During the first session (or an orientation session, if applicable), workshop leaders distribute a Participant Information Survey to each participant. The completion of these brief, 10-item surveys is optional and is not required for workshop participation. Completed surveys are collected from the participants and sent along with the Workshop Information Cover Sheet and Attendance Log to a person responsible for entering the information into the national database. Expected turnaround time for receiving the forms is generally two weeks after the conclusion of a workshop. While the structure for data entry varies by state, typically either (a) all data entry take place at the state level or (b) responsibility for data entry is divided regionally, with staff from selected organizations entering data on behalf of their peers. Decisions as to which model to use are generally based on adequacy of staffing for data entry/monitoring (e.g., is there sufficient staff time at a state level to devote to this task and keep up with demand, or does this task need to be parsed out regionally?). An additional consideration is the overall program management model, as some states centralize program management at the state level, whereas others take a decentralized approach with each region acting semi-autonomously.

### Developing the national, online database

Prior to the ARRA CDSMP initiative, data were collected via paper forms, which were mailed to a centralized location and entered into an Excel spreadsheet. This spreadsheet was sent semi-annually by each grantee to a central repository, where the data were cleaned, analyzed, and shared back with AoA and their respective grantees. This system was rather burdensome with data transfers from multiple partners. There was also a considerable time lag that occurred between the submission of semi-annual data and the receipt of the analyzed data.

In 2010, with the advent of the ARRA CDSMP initiative and the major expansion, a more efficient data system was needed for tracking the national rollout of CDSMP and assessing whether initiative goals were being met. Thus, the National Council on Aging (NCOA), the designated resource center for chronic disease SM education programs, developed an online national database. A custom application was developed by NCOA on the Salesforce.com platform expressly for this purpose. Salesforce.com was selected for reasons that include NCOA’s experience developing other data collection systems on the platform, cost-efficiency, web-based access, and data security.

Presently, the database is available as a free resource for all states implementing these programs, regardless of funding source. Upon request, users receive login information from NCOA, and can then enter workshop and participant data from any computer with Internet access. No software is required. All data are available in real-time, and data from any of the suite of in-person Stanford University CDSME programs can be entered. The data are stored securely and are de-identified at the participant level. The database does not contain participant names. Each participant is assigned a random unique identifier and is linked to a workshop though a separate unique identifier. Database users must view a recorded training webinar prior to accessing the system. Technical support related to database utilization and data entry is available via NCOA. Regular quality control activities, such as identifying erroneous duplicate workshops, are performed by NCOA and its database management partner, Senior Services. In addition, other quality measures are built into the system, such as prompting users to review workshop records with issues of concern such as workshop start and end dates that are fewer than 6 weeks apart and participant ages that are younger than 18 years (the minimum recommended participant age).

In addition to the ability to easily enter workshop and participant data, users have access to a variety of standard reports to inform program management and enhance quality assurance. These reports can be filtered by elements such as date, county, and host organization and offer a comparison to state and national data. Beyond the standard reports that are available to all database users, NCOA staff can develop custom reports in response to information and data requests from AoA leadership and other key stakeholders.

### Utility of collected data

The NCOA and AoA, as well as other program funders and stakeholders, use the information from the various data collection tools for numerous reasons, including to (1) comply with reporting requirements mandated by the authorizing statutes; (2) collect data for performance measures used in the justification of the budget to Congress and by program, state, and national decision makers; (3) effectively manage the program at the federal, state, and local levels; (4) identify program implementation issues and pinpoint areas for technical assistance activities; (5) identify best practices in program implementation and building sustainable program delivery systems and to develop resources to enable current and future program implementers to learn from and replicate these practices; and (6) provide information for reports to Congress, other governmental agencies, stakeholders, and to the public about grantee progress.

The uniform collection of these data elements using a coordinated online system has great practicality and utility for reporting and providing real-time monitoring and feedback. Using these data, AoA can perform state-based performance comparisons related to delivery site engagement, participant reach, participant retention, and program embedment/sustainability. These data can also enable program fidelity assessments to rapidly identify technical assistance needs and/or correct program drift.

Furthermore, these data can be used to develop webinars and other resources for state grantees and their partners (for the purposes of training, technical assistance, and/or strategic development) as well as generate standard and customized reports so grantees can identify local successes and opportunities for improvement. More specifically, these reports have been (and continue to be) used for quality control (e.g., identifying workshops that offered a “session zero,” or introductory orientation session, to determine impact on participant retention), planning (e.g., identifying host organizations that are categorized as faith-based when looking to engage additional partners of this same type), and reach to a specific population (e.g., number of African American participants who indicate a diabetes diagnosis).

Beyond these data uses at a federal/national level, this uniformly collected data also provide great benefit to state grantees and their partners. These data serve to inform key stakeholders about progress and challenges, guide quality control and assurance efforts and forward planning, and help justify the need for, as well as attain, additional funding sources (through grant applications or other mechanisms). Furthermore, researchers have utilized these data to address a variety of topics, including program participation of older adults with diabetes (17).

## Conclusion

Data collection for CDSMP and the suite of other Stanford University CDSME programs has been essential to nationwide program success and sustainability. With the inception of this database, states and community-based organizations offering CDSME had immediate, real-time access to their workshop and participant data for the first time. This proved to be an incredible value-add for the network. Not only does the data highlight program reach and inform program planning, it is also critical in terms of attaining additional resources to support implementation and infrastructure at national, state, and local levels. It is evident that the benefit gained from a national data collection system is certainly worth the investment in development, training, and maintenance. Future grand-scale initiatives delivering evidence-based programs are encouraged to use this ARRA CDSMP experience when creating data collection and monitoring systems.

## Conflict of Interest Statement

The authors declare that the research was conducted in the absence of any commercial or financial relationships that could be construed as a potential conflict of interest.

This paper is included in the Research Topic, “Evidence-Based Programming for Older Adults.” This Research Topic received partial funding from multiple government and private organizations/agencies; however, the views, findings, and conclusions in these articles are those of the authors and do not necessarily represent the official position of these organizations/agencies. All papers published in the Research Topic received peer review from members of the Frontiers in Public Health (Public Health Education and Promotion section) panel of Review Editors. Because this Research Topic represents work closely associated with a nationwide evidence-based movement in the US, many of the authors and/or Review Editors may have worked together previously in some fashion. Review Editors were purposively selected based on their expertise with evaluation and/or evidence-based programming for older adults. Review Editors were independent of named authors on any given article published in this volume.
